# Prospective Association Between Video and Computer Game Use During Adolescence and Incidence of Metabolic Health Risks: Secondary Data Analysis

**DOI:** 10.2196/44920

**Published:** 2023-07-06

**Authors:** Stephanie R Lebby, Sangeetha Shyam, Amutha Ramadas, Andrew R Bohm, Julia C Hill, Karen L Fortuna, Stephanie R Zoltick

**Affiliations:** 1 College of Nursing and Health Sciences University of Vermont Burlington, VT United States; 2 Centre for Translational Research Institute for Research, Development, and Innovation International Medical University Kuala Lumpur Malaysia; 3 Department of Biochemistry and Biotechnology Human Nutrition Unit Rovira i Virgili University Reus Spain; 4 Jeffrey Cheah School of Medicine & Health Sciences Monash University Malaysia Selangor Malaysia; 5 The Dartmouth Institute for Health Policy and Clinical Practice Geisel School of Medicine at Dartmouth Lebanon, NH United States; 6 Dartmouth College Hanover, NH United States; 7 Department of Psychiatry Geisel School of Medicine at Dartmouth Lebanon, NH United States

**Keywords:** video games, obesity, pediatrics, computer games, portable device, teenager, adolescents, health data, BMI, diabetes, high blood pressure, high cholesterol, metabolic disorder

## Abstract

**Background:**

Video and computer games are popular activities, with 72% of adolescents aged 13 to 17 years reporting video game use on either a computer, game console, or portable device. Despite high levels of video and computer game use in adolescence, relatively little scientific literature exists examining the association and effects of video and computer games on adolescents.

**Objective:**

The objective of this study was to examine the prevalence of video and computer game use among US adolescents and rates of positive screens for obesity, diabetes, high blood pressure (BP), and high cholesterol.

**Methods:**

A secondary data analysis was conducted using the National Longitudinal Study of Adolescent to Adult Health (Add Health) data, including adolescents aged 12 to 19 years between 1994 and 2018.

**Results:**

Respondents (n=4190) who played the most video and computer games had a significantly (*P*=.02) higher BMI and were more likely to self-report having at least one of the evaluated metabolic disorders: obesity (BMI >30 kg/m^2^), diabetes, high BP (BP >140/90), and high cholesterol (>240). With increased video or computer game use, there was a statistically significant increase in high BP rates in each quartile, with those with more frequent use also having higher rates of high BP. A similar trend was observed for diabetes, though the association did not reach statistical significance. No significant association was observed between video or computer game use and diagnoses of dyslipidemia, eating disorders, or depression.

**Conclusions:**

Frequency of video and computer game use is associated with obesity, diabetes, high BP, and high cholesterol in adolescents aged 12 to 19 years. Adolescents who play the most video and computer games have a significantly higher BMI. They are more likely to have at least one of the evaluated metabolic disorders: diabetes, high BP, or high cholesterol. Public health interventions designed to target modifiable disease states through health promotion and self-management may support the health of adolescents aged 12 to 19 years. Video and computer games can integrate health promotion interventions in gameplay. This is an important area for future research as video and computer games are integrated into the lives of adolescents.

## Introduction

Video and computer games are popular activities, with 72% of adolescents aged 13 to 17 years reporting video game use on either a computer, game console, or portable device [1]. Despite high levels of video and computer game use in adolescence, relatively little scientific literature exists examining the association of the effects of video and computer games on health risks in adolescents. With video and computer games being traditionally sedentary behaviors, the increase in video and computer game use is concerning. However, sedentary behavior is one of the leading modifiable risk factors for noncommunicable diseases. As video and computer games are commonplace in daily life, more research is needed to assess the association between video and computer games and chronic health conditions.

Sedentary behavior throughout the lifespan increases one’s risk for all-cause and cardiovascular disease (CVD) mortality, incident CVD, and type 2 diabetes [2]. Sedentary behavior is also associated with multiple forms of cancer [2]. Sedentary behavior may be associated with disease states due to hemodynamic, inflammatory, and metabolic processes resulting in impaired arterial health [3]. Similarly, systematic reviews have focused on the relationship between screen time in general and children’s adiposity measures (ie, BMI, percentage body fat, and waist circumference). For example, a systematic review examining 40 studies from 2010 to 2017 shows a positive association of screen time (ie, time spent on screen devices) with adiposity in children and adolescents (5 to 19 years old) in 85% of the studies [4].

In addition to being associated with chronic health conditions, sedentary screen time is related to psychological conditions. In a study of youth aged 11 to 20 years (n=2282), sedentary screen time (ie, time spent doing sedentary screen-based activities) was associated with the severity of depression (β*=*.23*, P*<.001) and anxiety (β*=*.07, *P*<.01) [5]. In particular, video game playing (β*=*.13, *P*<.001) and computer use (β*=*.17, *P*<.001) were associated with more severe depressive symptoms, and video game playing (β*=*.11, *P*<.001) was associated with the severity of anxiety [5].

This study aimed to examine the prevalence of video and computer game use among US adolescents and rates of positive screens for obesity, diabetes, high blood pressure (BP), and high cholesterol. Video and computer games are defined as electronic games played on either a personal computer or a video screen. This does not include smartphone games. The research question that guided this secondary data analysis was the following: To what extent is the frequency of video games and computer game use associated with self-reported chronic health conditions (ie, conditions that last a year or more and require ongoing medical attention, or limit activities of daily living, or both)?

## Methods

### Overview

A secondary data analysis was conducted in 2022 using the National Longitudinal Study of Adolescent to Adult Health (Add Health) 1994-2018. Add Health was a longitudinal study of US adolescents aged 12 to 19 years, starting in the 1994-1995 school year. This cohort was interviewed in 5 waves between 1994 and 2018. Waves 1 through 5 were collected in 1994-1995, 1996, 2001-2002, 2008-2009, and 2016-2018, respectively.

### Data Collection

Data were collected from the Add Health longitudinal study, in which all respondents who recorded data for both wave 1 (1994-1995) and wave 5 (2006-2018) were considered eligible. Add Health data came from 80 high schools selected from a sample frame of 26,666. Prior to sampling, schools were sorted by size, school type, census region, level of urbanization, and percent White race. Of the 80 selected, 52 were eligible and agreed to participate. Participating high schools then identified their feeder middle schools, resulting in one school pair. Parental consent was required for students to participate in the study. Some schools required passive consent (assuming parents granted permission for students to participate unless otherwise indicated), and others required active consent (a parental signature had to be returned before a student could participate). Data were collected from respondents using a combination of physical measurements, biospecimens, and self-reported surveys.

Demographic information was collected from the master data set, and exposure and outcome-specific variables were recorded from the respective wave data sets. Video game use variables were collected from wave 1 to capture video and computer game use while in adolescence (all wave 1 data were collected when the respondents were in grades 7-12). Outcome variables were recorded in wave 5 (21-23 years later), including outcomes related to health conditions. Any chronic conditions reported in a previous wave were recorded in wave 5. This allowed for maximum follow-up time through adulthood to capture as many conditions as possible.

### Variables

Variables in the data set were identified as exposures (video or computer game frequency) and outcomes (health outcomes). Wave 1 data were used at the exposure, and wave 5 data were used at the outcome. The exposure variable included self-reported video or computer game play duration and was measured in hours per week. Outcome variables included obesity (BMI >30 kg/m^2^), diabetes, high BP, hyperlipidemia, depression, and eating disorders. Obesity was measured using weight and height to calculate BMI, with BMI >30 kg/m^2^ indicating obesity. Hyperlipidemia was measured using a biospecimen. High BP was measured using physical measurements. Diabetes, depression, and eating disorders were measured by a self-reported survey of whether or not each participant had been diagnosed with the disorder. Self-reported measures of diabetes, depression, and eating disorders have been found to be reliable and valid in other studies [6-8]. Additionally, lifestyle variables were assessed. Lifestyle variables included self-reported measures of dairy, fruit, vegetable, grain, and pastry intake, as well as smoking, drinking, drug, and exercise habits.

### Statistical Analysis

The exposure of interest was split into quartiles to better understand the effects of none, low, moderate, and high video game use. All those in the lowest quartile were nonusers, and those in the remaining quartiles were evenly distributed. All baseline and demographic features listed above were evaluated across all quartiles for baseline differences in video and computer game use at the start of the study. Similarly, lifestyle characteristics were also evaluated across all quartiles at baseline. The relationship between video or computer game use at enrollment (baseline: wave 1) and reported metabolic and health outcomes at wave 5 was examined using logistic regression using the following four models: (1) the first model was an unadjusted crude model; (2) the second model adjusted for sociodemographics (ie, age, race, and sex); (3) the third model, in addition to sociodemographics, adjusted for socioeconomic characteristics (ie, public assistance and money for bills); and (4) the final model adjusted for sociodemographics, socioeconomic characteristics, and current lifestyle behaviors (ie, cigarette smoking, alcohol consumption, sedentariness, and fast food intake at wave 5), accounting for sampling weight and cluster sampling. Additionally, the exposure variable (duration of video or computer game played) was investigated as both a categorical value and a continuous variable in all models. All data were analyzed in 2022.

A supplementary analysis was carried out to evaluate the relationship between video or computer game use at enrollment (baseline: wave 1) and reported metabolic and health outcomes at wave 5 while adjusting for current BMI at wave 5 to account for the role of obesity in the development of other metabolic risks.

Only cases with complete data at both waves were used for statistical analysis, with no imputation of missing values required. A total of 6504 completed respondent records were identified in wave 1. Of these 6504 records, 6390 were completed through wave 5. Those respondents without complete wave data were removed from our analytic sample, for a total of 114 respondents (1.75%) at this point. Additionally, any records that contained incomplete data for any metric included in this study at any timepoint or wave were also removed. This removed 2200 respondents (33.83%) from our analytic sample. Due to a large amount of missing data across this longitudinal panel, we decided to use listwise deletion to avoid any bias or influence introduced with imputation. Our final analytic database included 4190 respondents.

Statistical analysis was performed using SPSS (version 25.0; IBM Corp).

### Ethical Considerations

This study did not require ethics approval as it involved secondary analysis of data available in the public domain.

## Results

### Video Games and Computer Game Use Among the Respondents

A total of 4190 respondents were included in this analysis. The median (range) hours spent by the respondents on video games and computer games was 1 (0-99) hours per week. Approximately 47% of the respondents did not play video games. The distribution of socioeconomic characteristics of respondents by quartile of the duration of video game played per week is shown in Table 1. Respondents who did not play any video games or computer games were typically older (16-17 years) and predominantly female (1440/4190, 72.5%) versus respondents in the other quartiles. While respondents who identified themselves as White were more likely to be represented in the lower quartiles (Q1 and 2), African American respondents had a higher likelihood of being in the higher quartiles (Q3 and 4) of duration spent playing video or computer games per week. A U-shaped association of the quartiles of the duration of video games and computer games played per week was observed with the location of residence and indicators of socioeconomic status, with urban residents and those receiving public assistance more likely to be in the extreme quartiles.

**Table 1:** Socioeconomic characteristics of all respondents by quartile of the duration of gameplay at enrollment. Values in the same row or category not sharing the same superscript are significantly different at *P*<.05 in the 2-sided test of equality for column proportions. Values with no superscript letters were not included in the test. Tests assume equal variances. Tests are adjusted for all pairwise comparisons within the rows in each subcategory using Bonferroni correction.

			Quartile of the duration of video games played per week								*P* value
			1 (n=1986)		2 (n=672)		3 (n=720)	4 (n=812)			
Duration of video games played (hours/week), median (95% CI)			0 (0-0)		1 (1-1)		2 (2-3)	7 (7-8)		<.001	
Age (years), median (95% CI)			16 (16-17)^a^		15 (15-16)^b^		15 (15-16)^b^	15 (15-16)^b^		<.001	
Age range (years)			13-18		12-18		12-18	13-18			
Male, %			27.5^a^		49.7^b^		52.1^b^	67.5^c^		<.001	
**Race, %**										<.001	
	White	68.7^a^		75.0^b^		66.1^a,c^		61.8^c^			
	Black or African American	20.4^a^		15.5^b^		20.8^a,b^		28.1^c^			
	American Indian or Native American	1.1^a^		1.9^a^		1.9^a^		1.4^a^			
	Asian or Pacific Islander	3.2^a^		4.0^a^		3.5^a^		3.2^a^			
	Other	6.5^a^		3.6^b^		7.6^a^		5.5^a,b^			
Completely urban residence, %			52.1^a^		44.9^b^		51.3^a,b^	52.2^a^		.01	
Receiving public assistance, %			8.5^a^		4.2^b^		7.7^a,b^	9.4^a^		.002	
Having enough money for bills, %			81.0^a^		84.8^a,b^		86.2^b^	82.3^a,b^		.01	

### Duration of Video or Computer Game Use and Lifestyle Characteristics

The distribution of lifestyle characteristics of respondents by quartile of the duration of video games played per week is shown in Table 2. Respondents in the higher quartiles of the duration spent on video or computer games more frequently reported consuming dairy products (54.6%); bread, pasta, or rice (65%); and pastry products (25.6%) on the previous day. They were also less likely to report not consuming fruit juice the previous day (49%). No association was found with vegetable consumption. Compared to those who reported playing video games, those who reported not playing them were more likely to report smoking (22%) and drug use (29%). No association was observed between video or computer gameplay and participation in a school club or physical education classes (proxy indicators of physical activity).

**Table 2:** Lifestyle characteristics of respondents by quartile of the duration of gameplay at enrollment. Values in the same row or category not sharing the same superscript are significantly different at *P*<.05 in the 2-sided test of equality for column proportions. Values with no superscript letters were not included in the test. Tests assume equal variances. Tests are adjusted for all pairwise comparisons within the rows in each subcategory using Bonferroni correction.

			Quartile of the duration of video game played per week								*P* value
			1		2		3		4		
**Ate dairy products yesterday**											<.001
	Did not eat	19.3^a^		14.3^b^		12.8^b^		15.0 ^b^			
	Ate once	35.0^a^		26.9^b^		31.5^a,b^		30.3^a,b^			
	Ate twice or more	45.6^a^		58.8^b^		55.7^b^		54.6^b^			
**Ate fruit or fruit juice yesterday**											.03
	Did not eat	23.6^a^		17.4^b^		20.8^a,b^		20.1^a,b^			
	Ate once	30.3^a^		32.6^a^		32.6^a^		30.9^a^			
	Ate twice or more	46.2^a^		50.0^a^		46.5^a^		49.0^a^			
**Ate vegetables yesterday**											.08
	Did not eat	32.5^a^		26.8^b^		31.4^a,b^		30.2^a,b^			
	Ate once	39.1^a^		44.0^a^		37.5^a^		40.9^a^			
	Ate twice or more	28.4^a^		29.2^a^		31.1^a^		28.9^a^			
**Ate bread, pasta, or rice yesterday**											.004
	Did not eat	8.9^a^		6.7^a,b^		7.1^a,b^		5.1^b^			
	Ate once	31.2^a^		27.4^a^		29.6^a^		30.0^a^			
	Ate twice or more	59.9^a^		65.9^b^		63.3^a,b^		65.0^a,b^			
**Ate pastry products yesterday**											<.001
	Did not eat	50.7^a^		42.0^b^		40.6^b^		41.7^b^			
	Ate once	30.6^a^		36.5^b^		37.2^b^		32.7^a,b^			
	Ate twice or more	18.7^a^		21.6^a,b^		22.2^a,b^		25.6^b^			
Regular cigarette smoking			22.0^a^		15.0^b^		16.8^b^		17.6^a,b^		<.001
Drink alcohol >2-3 times			59.4^a^		49.0^b^		50.1^b^		48.6^b^		<.001
Drug use			29.0^a^		22.2^b^		22.5^b^		23.5^b^		<.001
Participation in any sports-related clubs at school			100.0		100.0		100.0		100.0		N/A^c^
**Days or week of physical education classes**											.07
	0 days	43.7^a^		36.7^a,b^		34.57^a,b^		33.9^b^			
	1 day	1.5^a^		2.7^a,b^		4.4^b^		1.6^a,b^			
	2 days	5.4^a^		7.0^a^		6.8^a^		4.4^a^			
	3 days	9.6^a^		9.4^a^		8.4^a^		10.4^a^			
	4 days	1.7^a^		1.2^a^		1.2^a^		2.0^a^			
	5 days	38.0^a^		43.0^a,b^		44.6^a,b^		47.8^b^			

^c^N/A: not applicable.

### Duration of Video or Computer Game Use and Health

The distribution of metabolic and health indicators among respondents by quartile of the duration of video game played per week is shown in Table 3. Quartile 4 respondents had a significantly higher BMI and were more likely to report having at least one of the evaluated metabolic disorders (obesity (BMI >30 kg/m^2^), diabetes, high BP, and high cholesterol). The trend for an increased prevalence of high BP with increasing quartiles of video or computer game use was significant. A similar trend was observed for diabetes, though the association did not reach statistical significance. No significant association was observed between video or computer game use and the diagnosis of dyslipidemia, eating disorders, or depression.

**Table 3:** Metabolic and health indicators at wave 5 by quartile of the duration of gameplay at enrollment. Values in the same row or category not sharing the same superscript are significantly different at *P*<.05 in the 2-sided test of equality for column proportions. Values with no superscript letters were not included in the test. Tests assume equal variances. Tests are adjusted for all pairwise comparisons within the rows in each subcategory using Bonferroni correction.

	Quartile of the duration of video game played per week					*P* value
	1 (n=1986)	2 (n=672)	3 (n=720)	4 (n=812)		
BMI (kg/m^2^), median (95% CI)	28.4 (28.2-29.1)^a^	27.5 (27.3-28.3)^a^	28.1 (27.4-29.0)^a^	29.5 (29.1-30.1)^b^	<.001	
BMI >30 kg/m^2^, %	42.8^a^	37.2^a^	38.3^a^	47.1^b^	<.001	
Any one metabolic disorder, %	54.2^a^	50.7^a^	52.8^a^	60.3^b^	.001	
Ever diagnosed with diabetes, %	5.5^a^	5.2^a^	6.3^a^	7.4^a^	.20	
Ever diagnosed with high BP, %	16.8^a^	19.9^a,b^	21.7^b^	22.5^b,c^	.001	
Ever diagnosed with hyperlipidemia, %	16.6^a^	17.8^a^	17.7^a^	19.7^a^	.28	
Ever diagnosed with depression, %	26.8^a^	23.5^a^	23.8^a^	23.7^a^	.14	
Ever diagnosed with eating disorder, %	2.2^a^	1.2^a^	1.0^a^	1.5^a^	.08	

### Relationship Between Duration of Video or Computer Game Use and Health Indicators

In brief, the odds for the presence of any metabolic abnormality, obesity, or diabetes were significantly higher in the highest quartile compared to the lowest quartile of video and computer game use at enrollment. Quartile 4 (highest duration spent) compared to quartile 1 (lowest duration; this quartile reportedly did not play these games) had 23%, 27%, and 63% higher odds of reporting or having a metabolic abnormality, obesity, or diabetes, respectively.

#### Video or Computer Game Use and Metabolic Abnormalities

In the unadjusted model, every additional hour reportedly spent playing video games per week increased the odds of metabolic abnormalities by 1% (odds ratio [OR] 1.014, 95% CI 1.004-1.025). The relationship remained significant when adjusted for sociodemographic variables (age, sex, and race; OR 1.016, 95% CI 1.005-1.028) and additionally for socioeconomic indicators (OR 1.015, 95% CI 1.003-1.028). However, the association was not significant in the fully adjusted model.

#### Video or Computer Game Use and Obesity

In the unadjusted model, every additional hour reported to have been spent on playing video games per week increased the odds of obesity by approximately 1% (OR 1.013, 95% CI 1.003-1.023). When adjusted for sociodemographic variables (age, sex, and race), the strength of the association increased (OR 1.019, 95% CI 1.008-1.030). On additional adjustments for factors such as socioeconomic indicators, the association remained significant (OR 1.017, 95% CI 1.005-1.029). The relationship remained statistically significant even when the current lifestyle of the respondents and cluster sampling were accounted for (OR 1.016, 95% CI 1.003-1.029). However, such a significant association was not found with quartiles of video game playing duration in the final model.

#### Video or Computer Game Use and Diabetes

In the crude model, the duration of playing video games was not associated with diabetes incidence. However, when adjusted for sociodemographic variables (age, sex, and race; OR 1.020, 95% CI 1.004-1.037) and additionally for socioeconomic indicators (OR 1.019, 95% CI 1.002-1.037), the associations became significant. The association was attenuated and no longer statistically significant when the final model estimates were adjusted for current lifestyle (OR 1.011, 95% CI 0.991-1.031). Similarly, no significant association was found between quartiles of video game playing duration and the incidence of diabetes in the final model.

#### Video or Computer Game Use, High BP, and High Blood Cholesterol

The associations between video or computer game use, high BP, and high blood cholesterol were much less evident. While compared to those in Q1 (those who did not play the games), Q2, 3, and 4 had higher odds of reporting high BP or high blood cholesterol, a linear dose-response relationship was absent.

#### Video or Computer Game Use and Chronic Disease

Chronic disease was described as reporting any one of the following conditions: heart disease, kidney disease, stroke, and heart failure at wave 5. No association was observed between the risk for the prevalence of a chronic disease and the duration of video or computer game use (Table 4).

A supplementary analysis that adjusted for current obesity in evaluating the association between video or computer game use and cardiometabolic risks is presented in Multimedia Appendix 1. The results remained unaltered in this analysis.

**Table 4:** Association between duration of gameplay in adolescence (wave 1) and subsequent metabolic abnormality diagnosis (wave 5).^a^

Diagnosis and duration of video game use (hours/week)		Model 1	Model 2	Model 3	Model 4
**Any one of the metabolic disorders**					
	Quartile 1	1.000	1.000	1.000	1.000
	Quartile 2, OR (95% CI)	0.871 (0.731-1.038)	0.897 (0.745-1.080)	0.845 0.692-1.031)	1.348 (1.087-1.671)
	Quartile 3, OR (95% CI)	0.945 (0.797-1.121)	0.963 (.802-1.155)	1.015 (0.833-1.237)	1.111 (0.879-1.404)
	Quartile 4, OR (95% CI)	1.287 (1.090-1.519)	1.315 (1.094-1.580)	1.240 (1.018-1.510)	0.941(0.735-1.205)
	*P* value	.001	.002	.03	.70
	*R^2^*	0.005	0.017	0.013	0.063
	Continuous, OR (95% CI)	1.014 (1.004-1.025)	1.016 (1.005-1.028)	1.015 (1.003-1.028)	1.012 (0.998-1.026))
	*P* value	.006	.005	.01	.09
	*R^2^*	0.003	0.015	0.024	0.060
**Obesity**					
	Quartile 1	1.000	1.000	1.000	1.000
	Quartile 2, OR (95% CI)	0.792 (0.660-0.950)	0.856 (0.706-1.037)	0.818 (0.664-1.007)	0.736 (0.594-0.911)
	Quartile 3, OR (95% CI)	0.831 (0.697-0.990)	0.871 (0.722-1.050)	0.892 (0.729-1.092)	0.857(0.679-1.083)
	Quartile 4, OR (95% CI)	1.189 (1.008-1.403)	1.319 (1.098-1.585)	1.263 (1.037-1.539)	1.144 (0.897-1.460)
	*P* value	<.001	<.001	.002	.88
	*R^2^*	0.006	0.014	0.021	0.076
	Continuous, OR (95% CI)	1.013 (1.003-1.023)	1.019 (1.008-1.030)	1.017 (1.005-1.029)	1.016 (1.003-1.029)
	*P* value	.009	.001	.004	.02
	*R^2^*	0.002	0.011	0.019	0.073
**Diabetes**					
	Quartile 1	1.000	1.000	1.000	1.000
	Quartile 2, OR (95% CI)	0.792 (0.660-0.950)	0.856 (0.706-1.037)	0.818 (0.664-1.007)	1.155 (0.620-2.152)
	Quartile 3, OR (95% CI)	0.831 (0.697-0.990)	0.871 (0.722-1.050)	0.892 (0.729-1.092)	1.278(0.806-2.026)
	Quartile 4, OR (95% CI)	1.189 (1.008-1.403)	1.319 (1.018-1.585)	1.263 (1.037-1.539)	1.356(0.823-2.235)
	*P* value	<.001	<.001	.002	.46
	*R^2^*	0.006	0.014	0.021	0.023
	Continuous, OR (95% CI)	1.015 (0.999-1.031)	1.020 (1.004-1.037)	1.019 (1.002-1.037)	1.011 (0.991-1.031)
	*P* value	.06	.02	.03	.29
	*R^2^*	0.002	0.007	0.008	0.022
**High BP**					
	Quartile 1	1.000	1.000	1.000	1.000
	Quartile 2, OR (95% CI)	1.229 (0.983-1.537)	1.178 (0.926-1.499)	1.158 (0.892-1.504)	1.114(0.827-1.502)
	Quartile 3, OR (95% CI)	1.372 (1.109-1.698)	1.319 (1.048-1.661)	1.419 (1.108-1.817)	1.291(0.924-1.806)
	Quartile 4, OR (95% CI)	1.432 (1.169-1.755)	1.217 (0.967-1.531)	1.145 (0.891-1.471)	0.969(0.723-1.289)
	*P* value	.001	.09	.05	.92
	*R^2^*	0.006	0.023	0.027	0.047
	Continuous, OR (95% CI)	1.013 (1.002-1.024)	1.007 (0.995-1.019)	1.006 (0.993-1.019)	1.002(0.982-1.023)
	*P* value	.01	.27	.38	.84
	*R^2^*	0.002	0.021	0.024	0.044
**High blood cholesterol**					
	Quartile 1	1.000	1.000	1.000	1.000
	Quartile 2, OR (95% CI)	1.090 (0.865-1.373)	1.059 (0.827-1.357)	1.027 (0.785-1.343)	1.024(0.751-1.397)
	Quartile 3, OR (95% CI)	1.079 (0.861-1.351)	1.052 (0.825-1.339)	1.096 (0.845-1.420)	1.152)
	Quartile 4, OR (95% CI)	1.232 (0.998-1.520)	1.139 (0.900-1.441)	1.164 (0.904-1.498)	1.197(0.882-1.624)
	*P* value	.28	.76	.67	.76
	*R^2^*	0.001	0.012	0.013	0.017
	Continuous, OR (95% CI)	1.004 (0.992-1.016)	1.002 (0.989-1.016)	1.005 (0.991-1.018)	1.007(0.989-1.026)
	*P* value	.48	.73	.48	.44
	*R^2^*	0.000	0.011	0.012	0.017
**Chronic disease**					
	Quartile 1	1.000	1.000	1.000	1.000
	Quartile 2, OR (95% CI)	0.842 (0.441-1.610)	0.889 (0.442-1.785)	1.045 (0.499-2.191)	0.820(0.306-2.199)
	Quartile 3, OR (95% CI)	1.044 (0.583-1.870)	1.093 (0.583-2.050)	1.198 (0.606-2.366)	0.933 (0.432-2.014)
	Quartile 4, OR (95% CI)	1.529 (0.931-2.511)	1.493 (0.837-2.662)	1.538 (0.815-2.904)	1.050 (0.428-2.575)
	*P* value	.26	.46	.59	.06
	*R^2^*	0.05	0.008	0.023	0.050
	Continuous, OR (95% CI)	1.025 (1.005-1.044)	1.028 (1.007-1.048)	1.039 (1.014-1.066)	1.014(0.975-1.054)
	*P* value	.01	.007	.003	.48
	*R^2^*	0.006	0.012	0.041	0.052

^a^The multivariate logistic regression (enter) method was applied. Metabolic abnormality was defined as having been diagnosed with any one of the following: obesity, diabetes, high BP, and high cholesterol at wave 5. Metabolic disease was defined as having been diagnosed with any one of the following: heart disease, kidney disease, stroke, and heart failure at wave 5. Model 1 was an unadjusted model; model 2 was adjusted for demography (age, race, and sex); model 3 was adjusted for demography and socioeconomic characteristics (public assistance and money for bills); model 4 was adjusted for demography, socioeconomic characteristics, current lifestyle (cigarette smoking, alcohol consumption, sedentariness, and fast food intake at wave 5) and the clustering effect. No interaction or collinearity between covariates were found.

## Discussion

### Principal Findings

The objective of this study was to examine the prevalence of video and computer game use among US adolescents and assess its association with obesity and associated metabolic disorders longitudinally. Respondents who played the most video and computer games at enrollment had a significantly higher BMI and were more likely to report having at least one of the evaluated metabolic disorders (obesity, ie, BMI >30 kg/m^2^; diabetes; high BP; and high cholesterol) than those who did not report playing games over the 2 decades of follow-up. No significant association was observed for diabetes, hypertension, dyslipidemia, eating disorders, or depression.

The associations between video and computer game use and obesity are of public health importance. The raw mean difference in BMI between the lowest and highest quartiles of video game use was approximately 1 kg/m^2^. It is estimated that the odds of developing type 2 diabetes per unit increase in BMI (kg/m^2^) range between 1.19 and 1.38 [9]. Possible explanations for the relationship between video games and a significantly higher BMI include sedentary behavior, increased food intake during video game sessions, and insufficient sleep [10,11]. However, current literature suggests there is an inconsistent relationship between video games and obesity [12]. We acknowledge that obesity has a multifactorial etiology and requires a multipronged approach to effectively prevent and manage the epidemic in several populations [13,14]. The fully adjusted model evaluating the association between the duration of video game use (hours/week) and obesity accounts for approximately 7% of the variation in the outcome. While each factor may account for a small variance, identifying variables that are associated with the long-term development of obesity will aid in individualizing strategies for the prevention and treatment of obesity. Further research is needed on the role video games play in obesity-related behaviors.

It is noted that obesity usually precedes the development of metabolic abnormalities. This could explain why the association of video and computer game use with obesity was statistically significant, but the trend did not reach statistical significance for other metabolic disorders. The trend for an increased prevalence of high BP with increasing video or computer games aligns with current literature [15]. Elevated BP as an adolescent is associated with CVD outcomes as an adult [16]. Similar to the association between video games and BMI, video games may increase BP through increased food intake while playing and a lack of sleep [10,11]. Further, BP may be elevated due to the excitement, stress, and concentration needed for effective gaming [15]. Further research is needed on separating obesity-related behaviors from the stress-inducing behaviors of video and computer game play.

Similarly, there was an observed potential for an increased likelihood of diabetes, which did not reach statistical significance. Current literature suggests inconsistent findings exist on the relationship between video games and diabetes. One cross-sectional study of adolescents aged 14-18 years (n=307) found no association between video games and an elevated risk of diabetes [17], and another pilot study (n=12) found video games to induce a state of excitation sufficient to activate the sympathetic system and alter the course of glycemia in children with type 1 diabetes [18]. Similar to an increase in BP, excitation, leading to mental and physical stress, may contribute to the increase in blood glucose. Due to the inconsistency in the findings, more research is needed to determine the direction of the association between diabetes and video and computer games.

No significant association was observed between video or computer game use and the diagnosis of dyslipidemia, eating disorders, or depression. Current literature has mixed findings on the relationship between video and computer games and depression. One study (n=126) found mobile phone and TV viewing were associated with higher levels of depression a year later [19]. However, video game play was unrelated. Another study found video game playing (β*=*.13, *P*<.001) and computer use (β*=*.17, *P*<.001) were associated with more severe depressive symptoms [5]. No known studies are looking at the association of video game play with eating disorders (anorexia nervosa or bulimia). However, one study looks at the use of video games to treat bulimia. There are no studies currently associating dyslipidemia and video games, however, there is evidence that dyslipidemia is observed in obese and overweight youth [17].

### Limitations

The Add Health study is robust, yet the limitations of large databases and secondary data analyses are present. First, the quantification of exposures and outcomes is accurate within the limitations of the self-reported nature of this data. Second, the long retrospective period of this study is both to its benefit and a potential limitation. With more than 20 years between exposure (wave 1) and outcome (wave 5), there is adequate time to assess the development of the outcomes of interest into adulthood; however, this also opens the study up to more than 20 years of unmeasured confounding and loss to follow-up. However, this does not diminish the observed association. Third, in our categorization of video game use, quartile 1 included all respondents who reported that they did not play video games at enrollment. This approximately represented 48% of the respondents. The rest of the respondents were distributed between the remaining quartiles, resulting in a skewed distribution of respondents among the quartiles. We also acknowledge that the available data does not allow for a trajectory analysis of important confounders such as diet and physical activity. Thus, this analysis assumes that these variables were stable during the course of the follow-up and that residual confounding cannot be discounted. Finally, video game technology as well as social understanding around the topic has greatly changed in the past 20 years. These limitations, however, highlight areas for future study, as the vast majority of them are related to the secondary data source used. A purpose-built study design and data collection would address these limitations and help to examine the relationship found.

### Conclusions

The frequency of video and computer game use in adolescents aged 12 to 19 years is longitudinally associated with obesity, with trends for higher prevalence of diabetes, high BP, and high cholesterol after 20 years of follow-up. Adolescents who play the most video and computer games have a significantly higher BMI in adulthood. They are more likely to have developed at least one of the evaluated metabolic disorders: diabetes, high BP, and high cholesterol. Obesity, diabetes, high BP, or high cholesterol are all modifiable health conditions. As such, public health interventions designed to target modifiable disease states through health promotion and self-management may support the health of adolescents aged 12 to 19 years. Potentially, video and computer games can integrate health promotion interventions into gameplay. This is an important area for future research as video and computer games are integrated into the lives of adolescents.

## Appendix

Multimedia Appendix 1 Association between duration of gameplay in adolescence (wave 1) and subsequent metabolic abnormality diagnosis (wave 5), controlling for current BMI.

## Abbreviations

Add Health: Adolescent to Adult Health
BP: blood pressure
CVD: cardiovascular disease
OR: odds ratio
