# Supplementary material for: Prospective Association Between Video and Computer Game Use During Adolescence and Incidence of Metabolic Health Risks: Secondary Data Analysis
Source: JMIR Pediatr Parent. 2023 Jul 6;6:e44920. doi: 10.2196/44920 (PMC10360012; doi:10.2196/44920)
Supplement: Multimedia Appendix 1 [file pediatrics_v6i1e44920_app1.docx]

**Multimedia Appendix 1**

**Association between Duration of Gameplay in Adolescence (Wave 1) and Subsequent Metabolic Abnormality Diagnosis (Wave 5), Controlling for Current BMI**

| **Diagnosis** | **Duration of video game use (hours/week)** | **Model 5** |
| --- | --- | --- |
| Diabetes | Q1 | 1.000 |
|  | Q2 | 1.291 (0.657 – 2.535) |
|  | Q3 | 1.244 (0.768 – 2.015) |
|  | Q4 | 1.324 (0.804 – 2.182) |
|  | *P* value | 0.689 |
|  | R^2^ | 0.113 |
|  |  |  |
|  | Continuous | 1.011 (0.992 – 1.030) |
|  | *P* value | 0.266 |
|  | R^2^ | 0.112 |
|  |  |  |
| High blood pressure | Q1 | 1.000 |
|  | Q2 | 1.205 (0.890 – 1.631) |
|  | Q3 | 1.331 (0.941 – 1.881) |
|  | Q4 | 1.032 (0.771 – 1.382) |
|  | *P* value | 0.656 |
|  | R^2^ | 0.122 |
|  |  |  |
|  | Continuous | 1.001 (0.981 – 1.021) |
|  | *P* value | 0.916 |
|  | R^2^ | 0.119 |
|  |  |  |
| High cholesterol | Q1 | 1.000 |
|  | Q2 | 1.074 (0.785 – 1.468) |
|  | Q3 | 1.160 (0.830 – 1.620) |
|  | Q4 | 1.175 (0.863 – 1.599) |
|  | *P* value | 0.944 |
|  | R^2^ | 0.049 |
|  |  |  |
|  | Continuous | 1.007 (0.988 – 1.025) |
|  | *P* value | 0.479 |
|  | R^2^ | 0.048 |
|  |  |  |
| Chronic diseases | Q1 | 1.000 |
|  | Q2 | 0.847 (0.316 – 2.269) |
|  | Q3 | 0.929 (0.428 – 2.017) |
|  | Q4 | 1.050 (0.432 – 2.554) |
|  | *P* value | 0.058 |
|  | R^2^ | 0.053 |
|  |  |  |
|  | Continuous | 1.014 (0.975-1.054) |
|  | *P* value | 0.487 |
|  | R^2^ | 0.055 |

Model 5 = adjusted for demography, socio-economic characteristics, current body mass index, current lifestyle (cigarette smoking, alcohol consumption, sedentariness and fast food intake at Wave 5) and clustering effect.
